# Microfluidic sperm separation for intracytoplasmic sperm injection yields similar blastocyst rates compared with density gradient, however, may be of benefit for male factor infertility: a randomized sibling oocyte study

**DOI:** 10.1016/j.xfre.2026.08.003

**Published:** 2026-08-18

**Authors:** Rani Fritz, Alicia Arnott, Jessica Tozour, Linda Sung, Maria Saketos, Satu Kuokkanen, Vasilios Goudas, Anand Rajan

**Affiliations:** a Department of Obstetrics and Gynecology, Northwell Health Fertility, Manhasset, New York; b Department of Obstetrics and Gynecology, NYU Langone Reproductive Specialists of New York, Mineola, New York; c Department of Obstetrics and Gynecology, University of Pennsylvania, Philadelphia, Pennsylvania; d Department of Obstetrics and Gynecology, NYU Langone Long Island School of Medicine, Mineola, New York; e Department of Foundations of Medicine, NYU Langone Long Island School of Medicine, Mineola, New York

**Keywords:** Sperm separation, intracytoplasmic sperm injection, preimplantation genetic diagnosis

## Abstract

**Objective:**

To study whether microfluidic sperm separation in intracytoplasmic sperm injection cycles provides any benefit over density gradient sperm separation for good-quality embryo formation.

**Design:**

Randomized sibling oocyte study.

**Subjects:**

Patients undergoing intracytoplasmic sperm injection.

**Intervention:**

Microfluidic sperm separation vs. density gradient sperm separation.

**Main Outcome Measures:**

Primary outcomes were percentage of good-quality blastocyst formation. Secondary outcomes included sperm parameters after sperm separation, including percent motility, grade of forward progression, fertilization rates, blastocyst formation rates, and ploidy rates in the event that preimplantation testing for aneuploidy was performed. Additionally, subanalysis on those with male factor infertility was performed, evaluating primary and secondary outcomes above.

**Results:**

A total of 34 cycles enrolled into the study, yielding 456 oocytes, 231 randomized to microfluidics and 225 to density gradient (DG). Average microfluidic fertilization rate was 76.1% ± 19.2% and 72.4% ± 26.6% for DG. Microfluidic blastocyst formation rate was 58.4% ± 31.9% compared with 58.7% ± 30.5% for DG. Good-quality blastocyst formation rate for microfluidics was 51.4% ± 29.7% and for DG was 51.7% ± 30.9%. Euploid rates were 43.8% ± 35.5% in the microfluidics cohort and 33.7% ± 33.3% in the DG cohort. Subanalysis of cycles with male factor infertility revealed good-quality blastocyst formation rate was 45.9% ± 30.9% after microfluidics and 55.1% ± 29.2% for DG. Subanalysis of cycles with male factor infertility revealed an euploid rate of 50.0% ± 37.6% after microfluidics compared with 18.4% ± 24.5% for DG.

**Conclusion:**

There were similar rates of good-quality embryo formation after intracytoplasmic sperm injection using sperm separated with microfluidics compared with density gradient; however, subanalysis of the male factor infertility group revealed higher euploid rates with microfluidics.

**Trial Registration:**

This trial was registered in clinicaltrials.gov, ID number NCT04818593. URL: https://clinicaltrials.gov/study/NCT04818593. Date of registration: 3/25/2021. Date of first enrollment: 6/18/2021.

Sperm integrity and function are critical to embryo formation and in vitro fertilization (IVF) success rates. Male factor infertility accounts for approximately 1/3 of infertility diagnoses and is one of the most common diagnoses for IVF cycles in the United States. Of the millions of sperm that are produced in an ejaculate, a significant proportion of them are suboptimal (1). Although the traditional semen analysis identifies those with male factor infertility, factors beyond those evaluated with a traditional semen analysis, such as sperm deoxyribonucleic acid (DNA) fragmentation, may affect sperm quality and IVF success rates. Indeed, data have revealed that increased levels of DNA fragmentation have been found in infertile males with normal semen parameters (2). Furthermore, elevated levels of sperm DNA fragmentation, albeit inconsistent, have been associated with adverse fertilization rates, progression to good quality embryos, increased miscarriage rates, and adverse assisted reproductive technologies (ART) success rates (3, 4, 5, 6, 7, 8, 9, 10, 11).

Selection of healthy sperm for use in intracytoplasmic sperm injection (ICSI) is critical for ICSI success. Density gradient (DG) is a commonly used method to select sperm for ICSI, which depends on centrifugation. This technique requires mechanical and chemical processing that can increase oxygen free radical formation, possibly leading to sperm DNA damage and increased DNA fragmentation (12, 13). Microfluidic sperm separation (MFSS) has been proposed as a method to separate sperm in a fashion minimizing oxidative stress and adverse effects on sperm DNA (14, 15). Recently, ZyMōt Multi (850 ul) (Dx Now Inc., Gaithersburg, MD), an MFSS device, has been introduced in an attempt to separate healthier sperm without centrifugation. Studies evaluating sperm parameters and clinical outcomes using MFSS are limited. Sperm DNA fragmentation after ZyMōt MFSS separation has shown a significant reduction compared with DG and improved intrauterine insemination (IUI) outcomes (16, 17). A recent randomized controlled study evaluating ZyMōt MFSS compared with DG revealed no significant differences between high-quality embryo formation or ongoing pregnancy rates between the two sperm separation techniques; however, this study’s primary outcome was high-quality cleavage-stage embryo formation (18). Given the limited clinical data, we desired to further evaluate whether formation of high-grade embryos at the blastocyst stage would be significantly improved using the ZyMōt MFSS compared with DG for ICSI preparation using a sibling oocyte study model, which provides the optimal control group.

## Materials and methods

This study was approved by the institutional review board at the New York University (NYU) (IRB# S21-00021) and registered on clinicaltrials.gov (NCT04818593). Informed consent was obtained from both the patient and partner before enrollment in the study. We conducted a study randomizing sibling oocytes, evaluating ICSI outcomes after sperm separation using DG compared with ZyMōt Multi (850 μL) MFSS (ZyMōt Fertility, DxNow Inc.). Inclusion criteria included all couples in which both partners were over 18 years old, undergoing controlled ovarian hyperstimulation and oocyte retrieval with the intent or possible intent of undergoing ICSI insemination. Exclusion criteria included in vitro insemination (IVF), use of donor sperm, surgically retrieved sperm, less than six metaphase II oocytes retrieved, or sperm sample insufficient for use with the ZyMōt device, defined as surgically retrieved sperm, if the initial semen sample contained less than 0.5 million total motile sperm, or no sperm with observed progressive motility. Couples were approached for possible enrollment in the study by a physician in the practice if the couple intended to undergo IVF with the possibility of undergoing ICSI.

After controlled ovarian hyperstimulation and oocyte retrieval, in cycles in which six or more metaphase II oocytes were obtained, oocytes were randomized within the same cycle to be inseminated with sperm processed via DG and sperm processed with MFSS. It is a practice within our institution to write insemination orders as IVF, ICSI, or ICSI per embryologist. The decision as to whether to write the order as IVF, ICSI, or ICSI per embryologist is of the doctor of record, taking into account sperm parameters, previous IVF cycles, and other pertinent clinical information. In the event of ICSI per embryologist, the default is to perform IVF insemination unless sperm parameters on the day of oocyte retrieval reveal a possible advantage to ICSI insemination, determined by the embryologist. Therefore, patients who consented to the study and were written as ICSI per embryologist were made aware that, in the event of IVF insemination, they would be withdrawn from the study and the oocytes will only be inseminated via DG-processed sperm. Additionally, those undergoing ICSI, but with less than six metaphase II oocytes, were also withdrawn from the study and only inseminated via DG-separated sperm. In the event of an odd number of oocytes, the extra oocyte was inseminated via DG if the patient’s medical record number ended in an odd number, and MFSS if it ended with an even number.

The primary outcome of this study was to evaluate whether the percentage of good-quality embryo formation after ICSI was improved with the use of MFSS compared with DG. Good-quality embryos were defined as blastocyst-stage embryos on day 5 or day 6 of culture with an overall grade of good or fair according to the Society for Assisted Reproductive Technology (SART) grading system (19). Secondary outcomes were sperm parameters after sperm separation, including percent motility, grade of forward progression, fertilization rates, and ploidy rates in the event that preimplantation for aneuploidy (PGT-A) was performed. Fertilization rates were defined as the percentage of embryos with 2 pronuclei per metaphase II oocytes inseminated with ICSI. Blastulation rates were defined as the percentage of embryos reaching the blastocyst stage per fertilized oocytes. In the event of PGT-A, euploid rates were defined as the percentage of euploid embryos per embryos biopsied. Sperm parameters were evaluated manually by trained embryologists. Additionally, a subanalysis on those with male factor infertility was performed, evaluating primary and secondary outcomes above. Male infertility was defined by criteria published by the World Health Organization including sperm concentrations below 15 million/mL, total motility below 40%, and Kruger morphology below 4% (1).

### Controlled ovarian hyperstimulation

Controlled ovarian hyperstimulation using gonadotropins (Gonal-F, EMD-Serono, Rockland, MA; Follistim, Organon, Jersey City, NJ; Menopur, Ferring, Saint-Prex, Switzerland) was performed as per the treating physician. The protocol was based on the patient’s ovarian reserve status determined by antimüllerian hormone levels and antral follicle count, age, body mass index, and previous responses to controlled ovarian hyperstimulation. Either a gonadotropin-releasing hormone (GnRH) agonist (leuprolide, Abbott Laboratories, Chicago, IL) or an antagonist cycle (0.25 mg Ganirelix acetate, Organon, or 0.25 mg cetrotide, EMD-Serono) protocol was used to prevent premature ovulation. The GnRH agonists were initiated in either the luteal phase of the previous cycle or with the start of ovarian stimulation. The GnRH antagonists were generally administered when the lead follicle had a mean diameter of 14 mm or more, or when the estradiol level was 400 pg/mL or more. Oocyte maturation was achieved by administration of either human chorionic gonadotropin (hCG) 10,000 units subcutaneously, GnRH agonist with 4 mg leuprolide acetate subcutaneously, or a combination of both when the mean diameter of the lead follicle was 20 mm or more. Oocyte retrieval was performed under transvaginal ultrasound guidance 36 hours after hCG or GnRH agonist administration.

### Density gradient sperm separation

Density gradient centrifugation was performed using a one-layer preparation of 90% Isolate (Irvine Scientific, Santa Ana, CA) in 15 mL conical tubes. Semen was layered over 1 mL of gradient and then centrifuged for 15 minutes at 300 × g. The supernatant was removed and discarded. The sperm pellet was washed by mixing with Multipurpose Handling Medium Complete (Irvine Scientific) and centrifuging the sample for 5 minutes at 400 × g. After the wash, the supernatant was removed and discarded, and the pellet was resuspended in culture medium (Continuous Single Culture-NX Complete, Irvine Scientific), assessed for sperm parameters, and held at room temperature until insemination.

### ZyMōt microfluidic sperm separation

A total of 850 μL of raw semen was directly deposited into the inlet port of the ZyMōt 850 μL multidevice, followed by placement of 750 μL culture medium (Continuous Single Culture-NXcomplete, Irvine Scientific) in the outlet port and throughout the upper collection chamber. The device was then incubated in a humidified 37 °C CO_2_ incubator for 30 minutes. During incubation, sperm swam through the microporous filter and into the upper collection chamber, where they were recovered via the outlet port. A total of 500 μL of the sperm sample was removed and placed in a separate tube for analysis and insemination. After sperm processing, sperm parameters were evaluated and recorded for each preparation, including percent motility and grade of forward progression.

If the physician ordered ICSI per embryologist, the decision to perform ICSI was a decision on the basis of the final prepared semen sample and/or other relevant clinical data. The decision to perform ICSI vs. IVF was made before stripping the oocytes for ICSI. If IVF was performed, the couple was excluded from the study and the entire cohort of oocytes was inseminated via IVF. Similarly, in the event that there were fewer than six mature oocytes at the time of ICSI, the oocytes were inseminated via DG-separated sperm and the couple was excluded from the study.

### ICSI

After oocyte retrieval, oocytes were denuded of their cumulus cells per standard protocol. If at least six mature oocytes (determined by visualization of a polar body) were present, half the oocytes were inseminated with sperm prepared via the MFSS device and half with sperm prepared with DG. Patients whose medical record number was even had the group of oocytes inseminated with sperm separated via the MFSS device recorded first in the electronic medical record gamete sheet (beginning with oocyte #1). Patients with an odd medical record number had the oocytes inseminated with sperm separated via DG recorded first in the electronic medical record gamete sheet (beginning with oocyte #1). Once the number of mature oocytes was determined, the embryologist performing the ICSI procedure randomly selected half the oocytes for insemination with MFSS-prepared sperm and half for insemination with DG-prepared sperm. In cases with an odd number of mature oocytes, the remaining egg was sorted according to the patient’s medical record number: if the number was even, the remaining egg was inseminated with MFSS-prepared sperm, if the number was odd, it was inseminated with DG-prepared sperm. Inseminated oocytes were grouped into separate wells of a LifeGlobal 4-well GPS dish (Cooper Surgical) by the sperm separation method, one well contained all oocytes inseminated with sperm separated by the MFSS device and a separate well containing all oocytes inseminated with sperm separated by DG centrifugation.

Fertilization assessment was performed between 16 and 18 hours after insemination, and normal fertilization was considered for visualization of exactly two pronuclei. Resulting embryos were then cultured in Continuous Single Culture Medium-NX Complete (Irvine Scientific) in separate, numbered droplets in LifeGlobal microdrop GPS dishes (Cooper Surgical). The numbers of the droplets correlated to oocyte/embryo number in the medical record gamete sheet and, thereby, also correlated to the type of sperm used for the insemination. Development of the embryos was assessed on culture days 5 and 6, and embryo stage and grade were recorded for each embryo as per the SART criteria (19). Given the way our laboratory functions and changes in coverage of embryologists during the week, the embryologist grading the blastocyst may have been the same or a different embryologist than the one who randomized the oocytes and performed ICSI. Therefore, the embryologist at the time of blastocyst evaluation may not have been blinded to which oocyte, and therefore resulting embryo, was inseminated with MFSS and DG.

If the patient planned to have embryo biopsy for PGT, this was performed before cryopreservation. Embryos reaching the blastocyst stage, displaying an inner cell mass, having an overall quality grade of good or fair and having a trophectoderm quality deemed suitable by the embryologist were eligible to undergo the biopsy procedure. A small hole was made in the outer shell of the embryo using the Hamilton Thorne Lykos laser. Several trophectoderm cells were aspirated into a biopsy pipette, and the sample was stretched to allow for visibility of the cell junctions. The cells in the biopsy pipette were removed from the embryo with 2–5 shots of the laser at cell junctions and gentle stretching. The removed trophectoderm cells were then transferred to a small test tube with a buffer solution and sent to Cooper Genomics for aneuploidy testing via next generation sequencing. Embryos were defined as euploid if <20% of DNA was aneuploid. Embryos were defined as aneuploid if >80% of DNA was aneuploid. Mosaicism was defined as between 20% and 80% aneuploid DNA.

### Statistical analysis

All data were deidentified and analyzed by a statistician. A sample size calculation was performed using a two-tailed McNemar’s test with an alpha level of 0.05 and a power of 80% to detect a difference in good-quality formation rate of 7% with the use of MFSS. Assuming a 48% good-quality blastocyst development rate and a 7% improvement in good-quality blastocyst development with MFSS, we calculated a total of 408 oocytes randomized to either DG or MFSS separation. Primary outcome was evaluated using generalized linear mixed models (GLMM). Sperm parameters after MFSS and DG sperm separation, as well as euploid rates in the event that PGT-A was performed after ICSI comparing sperm separation with MFSS or DG, was assessed using GLMM or cumulative logit mixed models (CLMM), as appropriate. A random effect for cycle-specific identifier and the subject-specific identifier was used to account for within-subject and within-treatment correlation between rates in the two groups. A separate analysis was conducted on cycles with male factor infertility. Data are reported as model estimates with corresponding 95% confidence intervals. For the GLM analyses, coefficient estimates represent the difference between DG and MFSS groups, with MFSS as the reference group. For the CLMM assessing ordinal outcomes, results are expressed as odds ratios, indicating the odds of achieving a higher outcome category with MFSS relative to DG. The *P*-values are generated from a Wald test on the individual model parameter. A result was considered statistically significant at *P*<.05. The difference in good-quality blastocyst development rate between the MFSS and DG separation groups was analyzed separately using a GLMM with random effects for cycle-specific identifier and subject-specific identifiers to account for within-subject and within-treatment correlations. Study sample characteristics were calculated (mean ± standard deviation or median [25th, 75th percentiles]) for continuous variables; frequency and percent for categorical data. Means for MFSS and DG outcomes were calculated after calculation of means for each individual cycle. All analyses were performed using R Version 4.5.1.

## Results

A total of 54 cycles from 49 couples consented to be enrolled in the study. After applying the exclusion criteria, a total of 34 cycles from 33 couples were enrolled in the study (Fig. 1). Eight cycles were not enrolled because of fewer than six mature oocytes retrieved, seven were excluded because of IVF insemination, one cycle was excluded because of sperm parameters below the threshold for use of MFSS, one cycle because of azoospermia on day of retrieval, two cycles were withdrawn because of retrieval occurring after completion of study, and one converted to an IUI because of low response. Demographics of those enrolled in the study are summarized in Table 1. Average age was 36.5 ± 3.9 years, average gravidity was 0.97 ± 0.9, average parity was 0.32 ± 0.5, and average AMH value was 3.3 ± 2.8 ng/mL. Etiologies of infertility are summarized in Table 1. Initial sperm volume was 3.0 cc ± 1.7 cc, initial concentration was 28.1 million/mL ± 27.4 million/mL, initial sperm motility was 40.1% ± 13.8%, and initial sperm forward progression was 2.1 ± 0.3. Baseline sperm parameters for the male factor infertility cohort revealed an average initial volume of 2.9 ± 1.8 cc, initial concentration of 25.9 ± 22.5 million/mL, initial sperm motility of 38.9% ± 13.8%, initial sample forward progression of 2.0 ± 0.3, and Kruger morphology of 2.7% ± 1.2% (Supplemental Table 1, available online). Postprocessing sperm parameters and blastocyst formation results are summarized in Table 2. After processing, MFSS motility was 95.1% ± 7.4% and DG motility was 63.5% ± 23.5% (*P*<.01). A total of 608 oocytes were retrieved, of which 456 were mature and randomized to sperm separation with MFSS vs. DG. A total of 231 oocytes were randomized to MFSS and a total of 225 to DG. Average MFSS fertilization rate was 76.1% ± 19.2% and 72.4% ± 26.6% for DG (*P* = .35). The MFSS blastocyst formation rate was 58.4% ± 31.9% compared with 58.7% ± 30.5% for DG (*P* = .69). Good-quality blastocyst formation rate for MFSS was 51.4% ± 29.7% and for DG was 51.7% ± 30.9% (*P* = .77).

**Figure 1:**
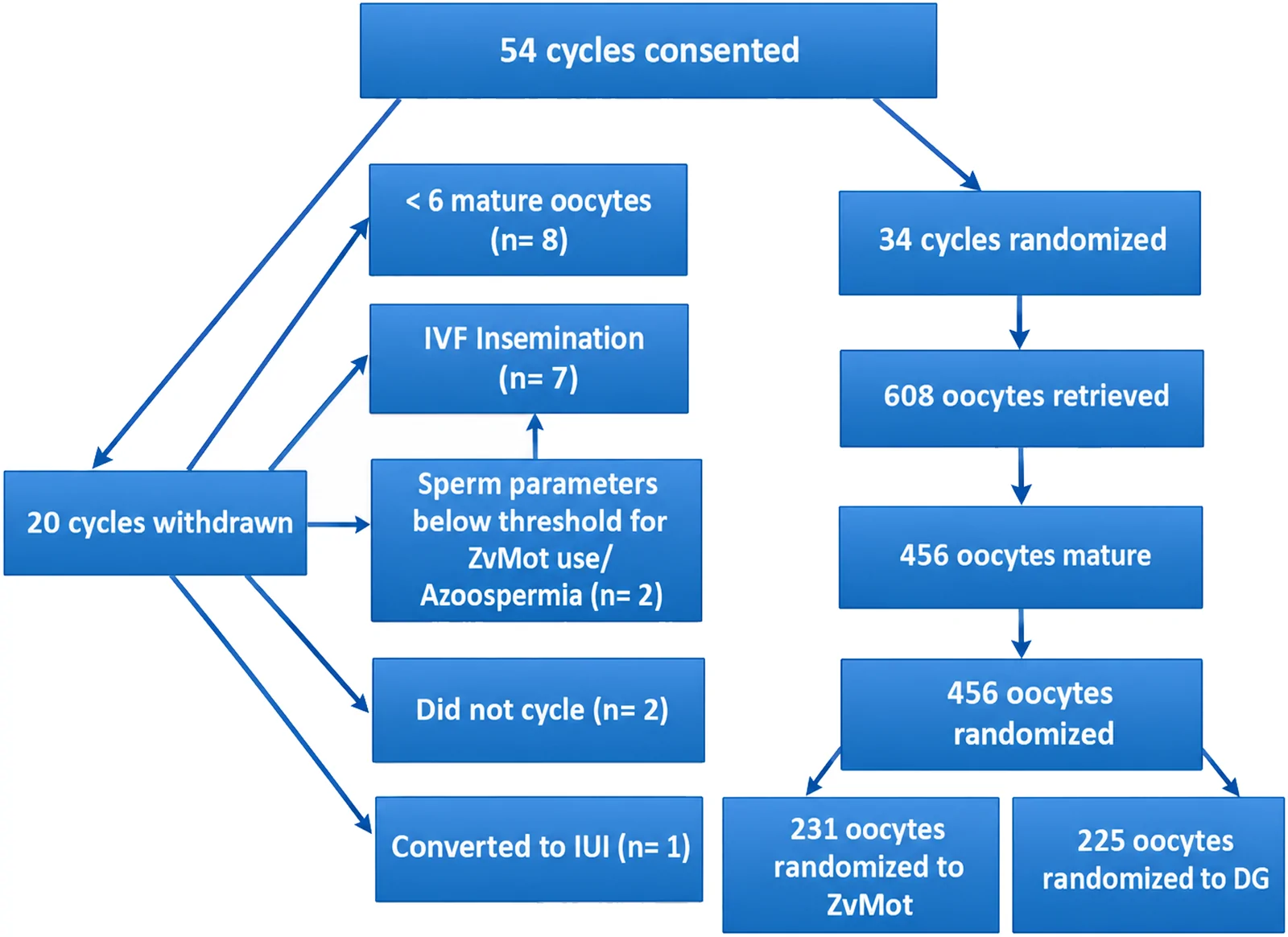
Flow diagram of cycle enrollment, exclusion, and randomization.

**Table 1:** Patient and cycle characteristics of those randomized into the study (n = 33 couples).

Age (y)	36.5 ± 3.9 y old
Gravidity (n)	0.97 ± 0.9
Parity (n)	0.32 ± 0.5
AMH value (ng/mL)	3.32 ± 2.8
Infertility diagnosis n (%)	
Male infertility (n)	21 (62%)
Oligospermia, severe (<5 million/mL)	2
Oligospermia, moderate (5–15 million/mL)	5
Asthenospermia (motility <40%)	2
Teratospermia (Kruger morphology <4%)	18
Endometriosis	2 (5.9%)
Tubal	2 (5.9%)
Uterine	0 (0%)
Ovulatory disorder	5 (15%)
Fertility preservation	0 (0%)
Gestational carrier	0 (0%)
Recurrent pregnancy loss	0 (0%)
Diminished ovarian reserve	10 (29.0%)
Unexplained	3 (8.8%)
Other	6 (18.0%)
First cycle n (%)	20 (58.8%)
Recurrent cycle n (%)	14 (41.2%)
Oocytes retrieved (n)	608
Oocytes maturity rate n (%)	456 (75.0%)
Initial semen volume (cc)	3.0 ± 1.7
Initial sperm concentration (million/mL)	28.1 ± 27.4
Initial sperm motility (%)	40.1% ± 13.8%
Forward progression (1–4)	2.1 ± 0.3

*Note:* Data are presented as mean ± standard deviation. AMH = antimüllerian hormone.

**Table 2:** Postprocessing sperm parameters.

	DG (225 mature oocytes from 34 cycles)	MFSS (231 mature oocytes from 34 cycles)	*P* value	Model estimate (95% CI)
Motility (%)	63.47 ± 23.48	95.11 ± 7.35	<.001	31.64 (24.41,38.87)
Forward progression (1–4) n (%)			<.001	59.12 (7.98,437.92)
2	6 (18)	5 (15)		
3	26 (76)	18 (53)		
4	2 (5.9)	11 (32)		
Fertilization rate (%)	72.44 ± 26.60	76.09 ± 19.20	.350	3.65 (−4.09,11.39)
Blastocyst formation rate (%)	58.73 ± 30.48	58.36 ± 31.94	.698	0.12 (−0.49,0.72)
Good quality blastocyst formation rate (%)	51.65 ± 30.86	51.42 ± 29.67	.773	0.09 (−0.52,0.70)

*Note:* Data are presented as raw numbers (mean ± standard deviation). Means for MFSS and DG outcomes were calculated after calculation of means for each individual cycle. CI = confidence interval; DG = density grading; MFSS = microfluidic sperm separation.

Of the blastocysts achieving good quality, 69 from the MFSS cohort were biopsied for PGT-A and 72 from the DG cohort. Table 3 summarizes ploidy rates on the basis of PGT-A results after MFSS and DG sperm separation. Euploid rate from the MFSS cohort was 43.8% ± 35.5% and 33.7% ± 33.3% for the DG cohort (*P* = .20). Aneuploid rate for the MFSS cohort was 39.3% ± 37.0 and 44.1 ± 36.3% for the DG cohort (*P* = .42). Mosaic rate for MFSS was 16.9% ± 27.4% and 25.3% ± 30.4% for the DG cohort (*P* = .34).

**Table 3:** Ploidy results for the subset of patients undergoing PGT-A.

	DG (72 embryos biopsied from 23 cycles)	MFSS (69 embryos biopsied from 24 cycles)	*P* value	Model estimate (95% CI)
Euploid rate (%)	33.66 ± 33.27	43.79 ± 35.50	.203	10.79 (−6.06, 27.64)
Mosaicism rate (%)	25.25 ± 30.44	16.94 ± 27.38	.342	−6.65 (−20.62, 7.32)
Aneuploid rate (%)	44.10 ± 36.31	39.27 ± 37.02	.422	−6.63 (−23.15, 9.89)

Male factor infertility subset	DG (33 embryos biopsied from 15 cycles)	MFSS (33 embryos biopsied from 14 cycles)	*P* value	Model estimate (95% CI)

Euploid rate (%)	18.41 ± 24.52	50.00 ± 37.55	.008	29.22 (8.35,50.09)
Mosaicism rate (%)	27.46 ± 35.97	17.86 ± 30.29	.466	−7.16 (−27.08, 12.77)
Aneuploid rate (%)	54.13 ± 35.97	32.14 ± 36.08	.044	−20.62 (−40.61,4)

*Note:* Data are presented as raw numbers (mean ± standard deviation). Means for MFSS and DG outcomes were calculated after calculation of means for each individual cycle. CI = confidence interval; DG = density gradient; MFSS = microfluidic sperm separation; PGT-A = preimplantation genetic testing for aneuploydy.

Subanalysis of cycles with male factor infertility (n = 21 cycles) was further analyzed (Supplemental Table 2). Average postprocessing MFSS motility was 96.9% ± 4.0%, and DG was 63.2% ± 24.1% (*P*<.01). Average fertilization rates postprocessing with MFSS were 78.1% ± 20.1% and DG were 66.3% ± 27.9% (*P* = .02). Average MFSS blastocyst formation rate was 51.9% ± 32.2% and DG 60.1% ± 28.7% (*P* = .64). Average good-quality blastocyst formation rate was 45.9% ± 30.9% post-MFSS and 55.1% ± 29.2% for DG (*P* = .70). Of the cohort with male factor infertility, 66 total embryos underwent PGT-A (Table 3). Euploid rate for MFSS (n = 20) was 50.0% ± 37.6% compared with 18.4% ±24.5% for DG (n = 9), *P*<.01. Aneuploid rate was 32.1% ± 36.1% for MFSS (n = 8) and 54.1% ± 35.9% for DG (n = 16), *P* = .04. Mosaicism rate for MFSS (n = 5) was 17.9% ± 30.3% compared with 27.5% ± 35.9% for the DG cohort (n = 8), *P* = .47.

## Discussion

This sibling oocyte study revealed that the ZyMōt MFSS device had similar good-quality embryo formation compared with DG. In the subanalysis of cycles with male factor infertility, our findings revealed a significant improvement in euploid rates, indicating a potential advantage of MFSS separation compared with DG in couples with male factor infertility; however, this result should be interpreted with caution because the subanalysis had a small sample size and a broad definition of male factor infertility was utilized, including men with isolated teratospermia. Centrosomes are organelles with multiple functions, one of which is microtubule formation and function during cell division (20). Centrosome contribution to the human zygote is paternally derived and responsible for the first meiotic division (21, 22). Data have shown that abnormal sperm parameters, including sperm DNA, adversely affect embryo ploidy rates after PGT-A (23, 24). It is possible that abnormal semen parameters, including increased sperm DNA fragmentation may lead to an increase in aneuploid rates related to compromised centrosome function and isolating healthier sperm may improve embryo ploidy rates.

A similar study by Kalyan et. al. (25) randomizing sibling oocytes to swim-up technique and MFSS revealed no differences in blastocyst development or pregnancy rates; however, this study differed in that the microfluidic chip evaluated differed from the one in this study, and the comparison group was swim-up as opposed to DG. Additionally, the blastocysts did not undergo PGT-A, and therefore no comparisons could be made for ploidy status. A sibling oocyte study by Meseguer et. al. (26) revealed a higher usable blastocyst rate; however, similar to the above study, it used a different microfluidic device and compared the device with swim-up as opposed to DG.

Similar to our findings, a randomized controlled trial by Quinn et al. (18) comparing embryo quality between MFSS with ZyMōt and DG revealed no significant differences between the two methods. Our study differed from the above study in that their primary outcome was cleavage-stage embryos, whereas in this study the primary outcome was blastocyst formation, which has been shown to increase live birth rates, particularly in good-prognosis patients (27). Additionally, our study randomized sibling oocytes, optimally accounting for confounding variables.

A similarly designed study by Godiwala et al. (28) evaluating euploid rates after PGT-A from sibling oocytes revealed no significant differences between MFSS and DG-separated sperm. Although these findings are similar to our findings, the differences were that in their subanalysis of male factor infertility, there were no differences in ploidy rates between DG and MFSS separated sperm. In both studies, the number of male factor infertility cycles was relatively small. Differences in these findings may be attributed to different criteria used to diagnose male factor infertility because the criteria were not specified in the study by Godiwala et al. (28). Furthermore, male factor infertility can vary greatly in severity and given the low cycle numbers of male factor infertility in our study, further analysis of varying degrees of severity would not be meaningful. Studies with a larger cohort of patients with male factor infertility are warranted. Lastly, the laboratories used to analyze ploidy status may have differed, as evident by large differences in reported mosaicism rates between both studies, which can affect reported ploidy (28).

The strengths of our study were the number of oocytes that were randomized as well as randomization of sibling oocytes, providing an additional barrier to confounding variables. A limitation of our study that must be emphasized was not evaluating for sperm DNA fragmentation, which has been shown to decrease following sperm separation with the ZyMōt MFSS device. A method to decrease DNA fragmentation may improve pregnancy rates and avoid invasive methods to treat high levels of DNA fragmentation, such as testicular sperm extraction (29, 30). Additionally, oocytes inseminated by IVF were also excluded from our study, thus limiting our findings to ICSI inseminated oocytes. The discrepancy between first (58%) vs. recurrent (42%) IVF cycles could have biased the data. Although our findings revealed improved euploid rates within the male factor infertility cohort, it is important to note that the degree of male factor infertility varied greatly, with the highest percentage revealing teratospermia. Additionally, multiple cycles from the same patient were included in the analysis, thereby potentially biasing the data because of overrepresentation from the same couple. Lastly, although embryos were graded using standardized grading systems, manual assessment by trained embryologists may introduce subjectivity into the grading process. Future studies using time-lapse imaging and/or artificial intelligence could limit the subjectivity of embryo grading.

## Conclusion

In conclusion, ZyMōt MFSS did not lead to improved blastocyst quality compared with DG, however, improved ploidy rates were observed after PGT-A in the subanalysis with male factor infertility. Although good-quality blastocyst formation rates were similar between the two sperm separation methods, MFSS may improve workflow in the embryology laboratory, but the potential additional cost for the device must be taken into account. Further, larger, and adequately powered studies are necessary to evaluate live birth rates and ploidy rates after PGT-A comparing ZyMōt MFSS sperm separation with DG in couples with male factor infertility.

## CRediT Authorship Contribution Statement

**Rani Fritz:** Writing – review & editing, Writing – original draft, Supervision, Project administration, Methodology, Investigation, Funding acquisition, Data curation, Conceptualization. **Alicia Arnott:** Writing – review & editing, Writing – original draft, Validation, Software, Resources, Project administration, Methodology, Investigation, Data curation, Conceptualization. **Jessica Tozour:** Writing – review & editing, Methodology, Data curation, Conceptualization. **Linda Sung:** Writing – review & editing, Methodology, Data curation, Conceptualization. **Maria Saketos:** Writing – review & editing, Methodology, Data curation, Conceptualization. **Satu Kuokkanen:** Writing – review & editing, Methodology, Data curation, Conceptualization. **Vasilios Goudas:** Writing – review & editing, Methodology, Data curation, Conceptualization. **Anand Rajan:** Writing – review & editing, Writing – original draft, Data curation.

## Declaration of Interests

R.F. served on the medical board for Cradle Genomics outside the submitted work and is also a member of ASRM Education Committee. A.A. has nothing to disclose. J.T. has nothing to disclose. L.S. has nothing to disclose. M.S. has nothing to disclose. S.K. has nothing to disclose. V.G. has nothing to disclose. A.R. has nothing to disclose.
