# Supplementary material for: Microfluidic sperm separation for intracytoplasmic sperm injection yields similar blastocyst rates compared with density gradient, however, may be of benefit for male factor infertility: a randomized sibling oocyte study
Source: F S Rep. 2026 Aug 18;7(5):378–85. doi: 10.1016/j.xfre.2026.08.003 (PMC13681311; doi:10.1016/j.xfre.2026.08.003)
Supplement: Supplementary file 1 [file mmc1.docx]

**Supplementary Table 1**

**Baseline Sperm and Parameters for Male Factor Patients**

| n=21 cycles |  |
| --- | --- |
| Initial Sample Volume (cc) | 2.9 ± 1.8 |
| Initial Sample Concentration (million/ml) | 25.9 ± 22.5 |
| Initial Sample Motility (%) | 38.9 ± 13.8 |
| Initial Sample Forward Progression  (1-4) | 2.0 ± 0.3 |
| Morphology, Kruger (%) | 2.7 ± 1.1 |

Data are presented as mean ± standard deviation.

**Supplementary Table 2**

**Post- processing Sperm and Cycle Parameters for Male Factor Patients**

|  | DG (119 mature oocytes from 21 Cycles) | MFSS (121 mature oocytes from 21 cycles) | p value (95% CI) | Model Estimate (95% CI) |
| --- | --- | --- | --- | --- |
| Motility (%) | 63.16 ± 24.06 | 96.86 ± 4.02 | <0.001 | 33.70 (23.90,43.51) |
| Forward Progression (1-4) n (%)  2  3  4 | 4(19)  17 (81)  0 (0) | 3(14)  14 (67)  4 (19) | 0.038 | 9.68 (1.13,83.08) |
| Fertilization Rate (%) | 66.33 ± 27.86 | 78.11 ± 20.10 | 0.022 | 11.78(1.82,21.73) |
| Blastocyst Formation Rate (%) | 60.12 ± 28.71 | 51.87 ± 32.21 | 0.640 | 0.14(-0.47,0.76) |
| Good Quality Blastocyst Formation Rate (%) | 55.08 ± 29.15 | 45.98 ± 30.86 | 0.703 | 0.10 (-0.41,0.60) |

Data are presented as mean ± standard deviation. Means for MFSS and DG outcomes were calculated following calculation of means for each individual cycle.
